# Mapping of the myxomatous mitral valve: The three-dimensional extension of mitral annular disjunction in surgically repaired mitral prolapse

**DOI:** 10.3389/fcvm.2022.1036400

**Published:** 2022-11-29

**Authors:** Raoul Biondi, Sophie Ribeyrolles, Christelle Diakov, Nicolas Amabile, Gabriella Ricciardi, Nizar Khelil, Alain Berrebi, Konstantinos Zannis

**Affiliations:** ^1^Department of Cardiovascular Surgery, Institut Mutualiste Montsouris, Paris, France; ^2^Department of Cardiology, Institut Mutualiste Montsouris, Paris, France; ^3^Department of Cardiac Surgery, Centre Hospitalier Universitaire Lille, Institut Pasteur de Lille, Lille, France

**Keywords:** 3D transesophageal echocardiography, mitral annular disjunction, mitral regurgitation, mitral valve prolapse, mitral repair

## Abstract

**Objectives:**

This study aimed to describe the heterogeneous extension of mitral annular disjunction (MAD) and assess the hypotesis that different phenotypes of disjunction are not associated with increased surgical challenges.

**Background:**

Mitral regurgitation (MR) is the most common end-stage scenario of degenerative mitral valve disease (DMVD). Few data exist on the three-dimensional extension and geometry of MAD, as well as for its role in valvular dynamic and coaptation.

**Methods:**

A total of 85 consecutive subjects, who underwent elective mitral valve repair (MVR) for MMVD at our Institution between November 2019 and October 2021, were studied retrospectively. The extension and geometry of MAD was assessed using the digitally stored volumetric datasets of real-time 3D transesophageal echocardiography (TEE). Annular phenotypes and surgical repair techniques were analyzed.

**Results:**

Mitral annular disjunction was diagnosed in 50 out of 85 patients (59%) with Barlow disease (BD). A detailed analysis of MAD extension was conducted on 33 patients. Two pattern of disjunction were identified: a bimodal shape was highlighted in 21 patients, while a more uniform distribution of the disjuncted annulus was observed in 12 patients. The bimodal pattern was characterized by lower disjunction distance (DD) at the 140°–220° arch (3.6 ± 2.2 mm), while a more regular DD was measured in the remaining patients. All patients successfully underwent MVR. Triangular leaflet resection was performed in 58% of the cases, neochordae implantation in 9%, and notably a 27% received an isolated annuloplasty.

**Conclusion:**

Rather than a binary feature, MAD should be taken into account in its complex and heterogeneous morphology, where two major phenotypes can be identified. Despite its anatomical complexity, MAD was not associated with an increased surgical challenge; conversely a peculiar subgroup of patient was successfully treated with an isolated annuloplasty.

## Graphical Abstract

Mitral annular disjunction (MAD) was analyzed and measured in thirty-three patients who underwent mitral valvular repair due to severe regurgitation. Our analysis showed that central regions of the posterior mitral annulus (MA) are less frequently the most disjuncted (22%). Nevertheless, up to date, MAD is still commonly diagnosed and measured in parasternal long axis view. If corroborate by further evidence, transesophageal echocardiographic (TEE) should considered as modality of choice for the diagnosis of MAD presence over the long-axis transthoracic evaluation, in order to avoid any diagnostic bias. 
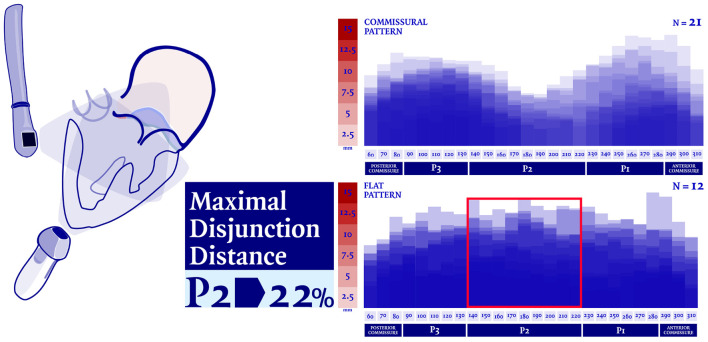

## Introduction

Diffuse myxomatous mitral valve degeneration (MMVD) and fibro-elastic deficiency are the two main histological phenotypes of degenerative mitral valve disease (DMVD), which accounts for an estimated worldwide burden of 18.1 million of cases in 2017 (1). Mitral regurgitation (MR)–due to mitral valve prolapse (MVP), chordal alterations, annular enlargement and/or abnormalities–is the most common end-stage scenario of DMVD. Although usually referred to as a simple displacement of the mitral annulus (MA), characterized by the posterior leaflet attachment on the atrial wall, mitral annular disjunction (MAD) is actually a structural variation of the atrioventricular junction, in which annular tissue is “stretched” and extends its roots into both atrial wall and the left ventricular (LV) musculature. Although passively driven by the systolic contraction, it is well established that the coupled motion between MA and left ventricle is pivotal to reach a balanced distribution of the stresses normally applied on the mitral complex during each cardiac cycle. In the context of a relatively normal LV function, assessed by both ejection fraction and strain, Lee et al. (2) characterized for the first time the 3-dimensional extension of MAD, correlating its paradoxical systolic dilatation with a decoupled annular-ventricular function. The decoupling between the LV contraction and the annular motion, which follows instead the atrial wall motion as a consequence of the disjunction, is the first insight and quantitative evidence provided regarding the functional implication of mitral disjunction in valvular dynamics. Likewise, the disjuncted MA is a peculiar entity which increases the 3-dimensional complexity of the native mitral valve. The posterior atrioventricular junction in the non-disjuncted annulus is commonly considered as the point where the leaflet, the atrial wall, the ventricular musculature and the fibro-adipose groove tissue converge, which corresponds echographically to the leaflet hinge point. When MAD occurred, this structure–which is indeed annular tissue (3)–evolves from the well-recognized yet complex saddle-shape ring toward an even more complex 3-dimensional surface. The gap between the hinge line of the posterior leaflet and the crest of the LV, measured in parasternal long axis view, is used to discriminate the presence of disjunction and reported as MAD length. Characterizing the disjunction as a binary variable is an incomplete approach, although fair in epidemiological terms and when arrhythmic risk stratification is needed (4). Anyhow, an irregular distribution pattern of the annular disjunction was pointed out in the works of both Lee et al. (2) and Toh et al. (5). A theoretical contribution to the disjunction dilemma has been recently brought by Faletra et al. (6). In the attempt to clarify the anatomical substrate of the disjunction, the authors postulated the existence of two different entities: a misinterpreted entity, called pseudo MAD in authors’ words, in which the portion of the billowed posterior leaflet which is closer to the MA is pushed against the left atrial wall through the systole, and a true MAD, in which a clear displacement of the hinge point toward the atrium is recognizable through all the cardiac cycle. With this study we aim to describe the heterogeneous extension of MAD, to explore the relationship between different MAD extension and surgical techniques and to support the research on the underlying pathology and dynamic of DMVD.

## Materials and methods

### Study population

A total of 85 consecutive subjects, who underwent elective mitral valve repair (MVR) for myxomatous degeneration at our Institution between November 2019 and October 2021, were studied retrospectively. All patients underwent 2-dimensional and 3-dimensional transesophageal echocardiographic (TEE) examination using a standardized protocol for the preoperative evaluation of the leaflets and annular pathology, as well as the quantification and description of the regurgitation. MMVD was defined by the presence of excess leaflet tissue and leaflet thickening greater than 5 mm, with a resulting prolapse of at least 2 mm into the left atrium on the parasternal long axis view (7). Patients were excluded in presence of any of these conditions: fibroelastic deficiency phenotype of the mitral valve, any degree of mitral stenosis, any degree of aortic valve disease, concomitant ischemic disease, pericardial disease, congenital heart disease, endocarditis, cardiomyopathy, or previous cardiac surgery.

### Imaging

**FIGURE 1:**
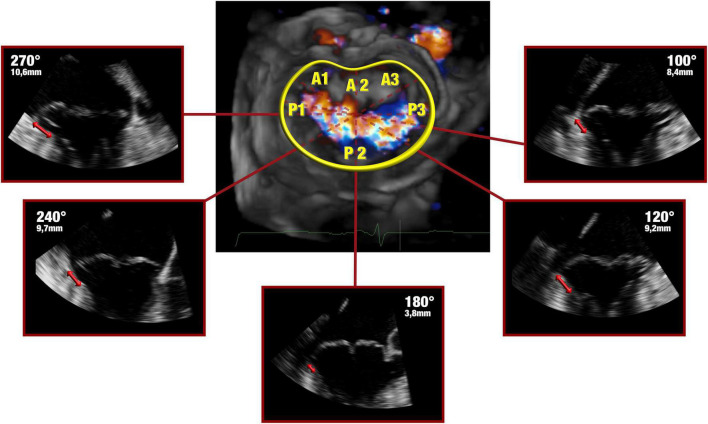
Late systolic frames of the digitally stored datasets were analyzed offline. The 3-dimensional anatomy of MAD was assessed by rotating a plane orthogonal to the ideal annular plane at 10° intervals, as already described by Lee et al. (4). Bidirectional red arrows highlight the extent of the disjuncted annulus.

Comprehensive real time 3-dimensional TEE was performed with an EPIQ CVx ultrasound system (Philips Healthcare, Andover, MA, USA) equipped with an xMATRIX array transducer X8-2t (Philips Healthcare, Andover, MA, USA). Valvular measurements and the volumetric datasets were acquired by trained cardiologists in both pre-operative and peri-operative assessment of the valvular pathology. During intraoperatively evaluation, patient breath-holding was performed to avoid stitching artifacts. The morphological features of the myxomatous mitral valve were assessed and analyzed using a semi-automatic dedicated quantification software (Mitral Valve Navigator, Philips Healthcare). To begin with, in the late-systole, four reference points were selected and tagged (anterior and posterior mid-point of the echographic annulus along with anterolateral and posteromedial segments); in addition, a manual outline of the annular perimeter was performed when adjustments were needed, in order to optimize the position in the intermediate reference points. Furthermore, the commissures were marked in the short axis plane. As final step, the shape of both mitral leaflets and their coaptation were traced in multiple planes from commissure to commissure. A 3D-rendered surface was then generated as topographical reconstruction of the valve, and some key parameters were measured and taken into account to characterize the mitral valve, namely: the anteroposterior diameter, the commissure width, the circumference, the tridimensional annular area and height (defined as the maximal vertical distance between the highest and lowest point of the geometric saddle shape), the leaflets’ total area and the total prolapsing or billowing volume. Surrogate equations were used to convey annular ellipticity and non-planarian saddle-shape of the valve: the ratio between the anteroposterior diameter to commissure width and the annular height to commissure width, respectively. The ratio between the total leaflet area and the annular area was additionally calculated as a measure of leaflets’ redundancy and prolapse. As the morphological and geometric reconstruction of Mitral Valve Navigator is founded on the semi-automatic detection of the leaflets’ profile and hinge point, the data on the 3-dimensional anatomy of the disjuncted annulus were obtained from the analysis of the same digitally stored volumetric datasets with a second dedicated software (QLAB, Philips Healthcare). The presence, extension and distribution of MAD were then assessed on the same frame chosen for the Mitral Valve Navigator’s rendered reconstruction. Three orthogonal imaging planes were adjusted to ensure that the long axis planes bisected the aortic and mitral valves, while the short axis plane was set parallel to the plane of the latter. As already described by Lee et al. (2), the presence and extension of MAD were analyzed and measured in radial planes rotated around the long axis at 10° intervals Figure 1. In order to pay careful attention to a lesser evident separation (<5 mm) between the leaflet hinge point and the LV free wall, and to differentiate a true disjunction from the systolic apposition of the billowed leaflet (6), a careful frame-to-frame analysis was conducted.

### Statistical methods

Data are expressed as mean ± standard deviation (SD), median (interquartile range), or percentage, as appropriate. All morphological valvular data were indexed to the body surface area or the end-diastolic volume (EDV), when appropriate. Furthermore, disjunction distance (DD) was indexed to the annular circumference and the statistical analysis was conducted on both individual and indexed values. Patients underwent two main categorizations based on anatomical MAD distribution or surgical mitral repair techniques. Differences across groups for baseline characteristics, mitral valve detailed anatomy, annular dimensions, leaflet morphology and operative data were determined by the use of chi-square test, Fischer exact test, Student’s *t*-test or by Mann–Whitney test as appropriate. The Shapiro–Wilk and Kolmogorov–Smirnov tests were used to analyze the normality distribution of continuous data. Prolapse volume and its ratio with EDV were not normally distributed when the anatomical patterns of disjunction were analyzed. On the other hand, the normality analysis of the surgical groups identified the need for a non-parametric analysis not only for prolapse volume and its ratio with EDV, but also for the indexed measures of commissure width, circumference, annular, and leaflet area as long as prosthetic ring size. All data analyses were performed using SPSS version 26.0 (IBM Corp., Armonk, NY, USA). A value of *p* < 0.05 was considered significant.

## Results

### Study population

In 11 out of 85 subjects, no evidence of MAD or signs of anomalous systolic thickening of the basal ventricular musculature was found. Of the remaining 74 subjects with MMVD and ventricular curling motion, 24 were found to show only a pronounced displacement of the basal portion of the posterior leaflet toward the atrial wall, a feature recognized as pseudo-MAD; therefore, they were excluded from this analysis. In 17 out of 50 cases, a precise measurement of the disjuncted annulus on the complete extent of the mitral circumference was not possible due to the presence of calcifications, artifacts or an incomplete acquisition of the volumetric datasets. True MAD was thus analyzed in 33 patients, whose characteristics are shown in Table 1. The mean age at the time of operation was 59.0 years, 54.5% of patients were men with a mean body surface area of 1.84 m^2^ and a low estimated operative risk (euroSCORE II: 1.4%).

**TABLE 1:** Patient characteristics.

		Distribution pattern		
		
	MAD (*n* = 33)	Commissural (*n* = 21)	Flat (*n* = 12)	*P*-value
**Clinical**				
Age, years	59.0 ± 12.5	61.6 ± 10.8	54.6 ± 14.4	0.11
Men	18 (55)	12 (57)	6 (50)	1.00
Body surface area, m^2^	1.90 ± 0.3	1.91 ± 0.3	1.90 ± 0.2	0.96
**Echocardiography**				
**Left ventricle**				
Ejection fraction, %	65.5 ± 5.4	65.3 ± 5.0	65.8 ± 6.4	0.84
End-diastolic volume, ml	141 ± 40	135 ± 35	154 ± 48	0.25
End-systolic volume, ml	48 ± 15	47 ± 14	51 ± 16	0.48
**Mitral valve**				
Number of prolapsed segment	1.27 ± 0.84	1.29 ± 0.78	1.25 ± 0.97	0.91
**Site of lesions**				
Isolated A1	0 (0)	0 (0)	0 (0)	
Isolated A2	0 (0)	0 (0)	0 (0)	
Isolated A3	0 (0)	0 (0)	0 (0)	
Isolated P1	0 (0)	0 (0)	0 (0)	
Isolated P2	13 (39)	9 (43)	4 (33)	
Isolated P3	2 (7)	1 (5)	1 (8)	
Bileaflet involvement	17 (52)	8 (38)	9 (75)	
**Commissure involvement**				
Anterolateral commissure	1 (3)	0 (0)	1 (8)	
Posteromedial commissure	3 (9)	1 (5)	2 (17)	
Chordal rupture	16 (48)	12 (57)	4 (33)	0.20

Values are mean ± SD or *n* (%). MAD, mitral annular disjunction.

### 3-dimensional geometry of disjunction

**FIGURE 2:**
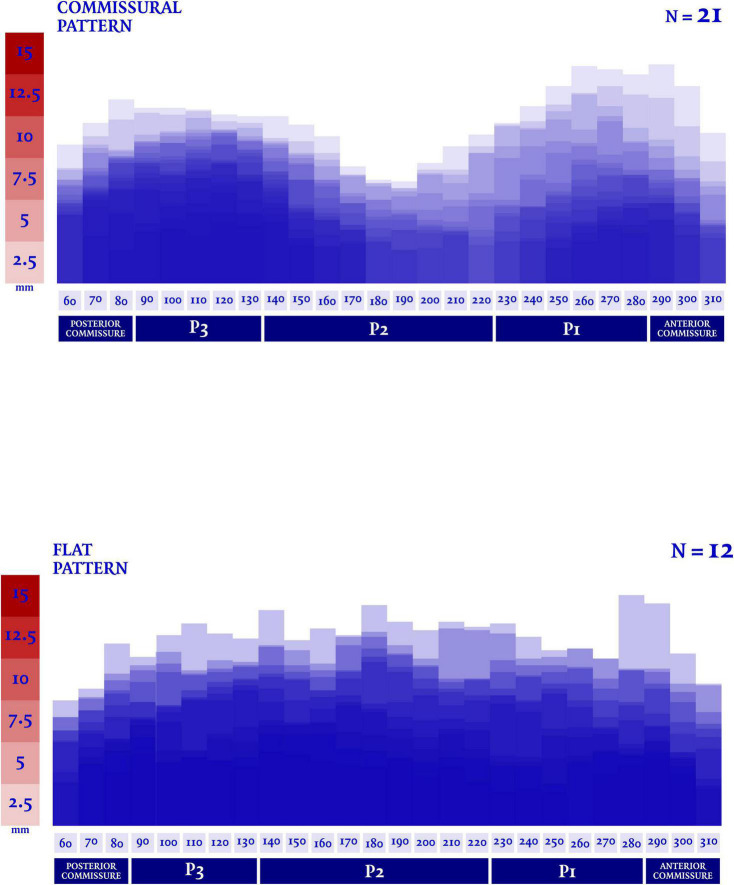
Disjunction distance (DD) was measured in multiple planes orthogonal to the ideal annular plane, rotating at 10° intervals. Each value was singularly plotted into a distribution map and then two patterns of disjunction were identified. A commissural pattern was recognized in 21 patients, conversely a flat pattern of disjunction was observed in 12 patients.

**FIGURE 3:**
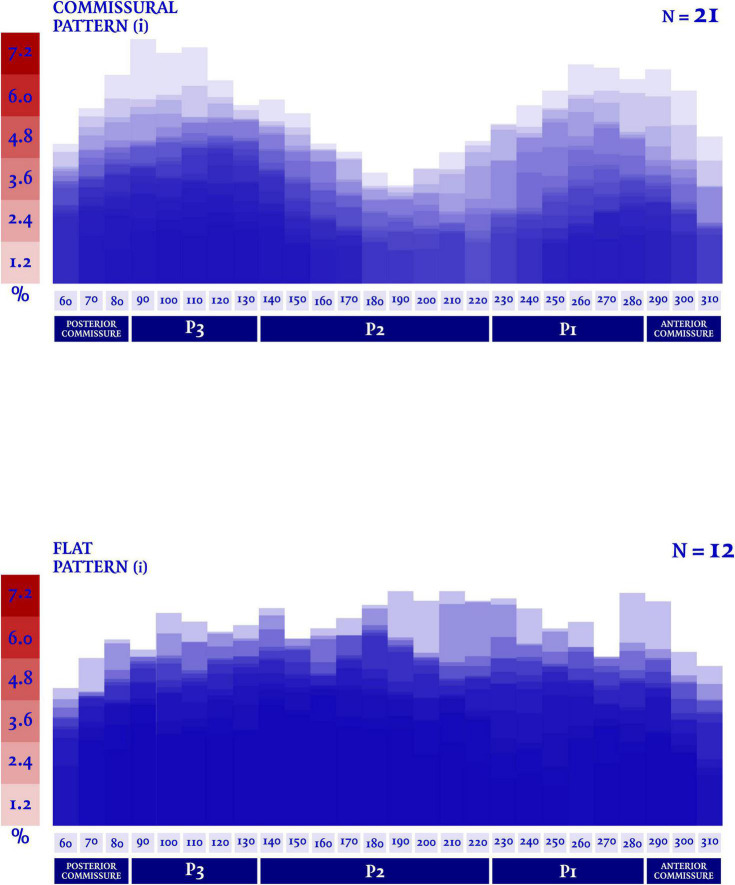
A dedicated quantification software (Mitral Valve Navigator, Philips Healthcare) was used for the rendering analysis of the valvular geometry. The circumferential values of disjunction distance (DD) were then singularly indexed to the total mitral circumference, in order to express the disjunction distance as a percentage of the total circumferential length of the valve.

In our analysis the extent of the myxomatous disease led to a prolapse of both anterior and posterior mitral leaflets in 17 cases (51.5% of the subjects), while a prolapse limited to the posterior leaflet was identified in 14 cases. In the 2 remaining cases, a prolapse of the posterior commissure was described preoperatively and confirmed after surgical inspection. The most frequently involved site of lesion was the P2 scallop, in 23 cases (69.7%). In almost half of these cases (*n* = 11, 47.8%), additional lesions were reported besides the P2 prolapse: a concomitant prolapse of P3 and P1 was identified on 7 and 4 patients, respectively. Additionally, in 3 cases an isolated prolapse of P3 scallop and the adjacent posterior commissure was identified. Sixteen cases (48.5%) of chordal rupture were identified, both after the preoperative echocardiography or after surgical inspection. In the context of a bileaflet prolapse, only 4 chordal rupture were reported (22.2%). Conversely, when the prevalent prolapse phenotype involved selectively the posterior leaflet, chordal rupture was reported in 10 cases (71.4%) and either the two cases of commissural prolapse were due to a chordal rupture. The maximal DD was located most frequently at P3 (*n* = 16). P2 (*n* = 7) and P1 (*n* = 6) were less commonly identified as the regions with the peak DD, such as the posterior and anterior commissure (respectively, the most disjuncted point in 3 and 1 cases). Interestingly, the maximal DD occurred in the prolapsing segment of the posterior mitral valve only in 13 cases out of 33 (39.4%). The circumferential values of DD were, then, singularly plotted into a distribution map. Two patterns of disjunction were identified: a bimodal shape was highlighted in 21 patients, while a more uniform distribution of the disjuncted annulus was observed in 12 patients, as shown in Figures 2, 3. Detailed regional values of disjunction are displayed in Table 2. The bimodal–“commissural”–pattern is characterized by an increased DD of the 90°–130° arch (7.2 ± 1.8 mm) as well as the 60°–80° (5.4 ± 2.2 mm) and 230°–280° arch (5.0 ± 3.4 mm), with lower values measured at the 140°–220° arch (3.6 ± 2.2 mm). The maximal disjunction is located at 120° on the ideal circumference of the valve, with a mean extension of 7.6 ± 1.8 mm. The extent of disjunction of P3 reaches a statistical significance threshold when compared to all the other segments. The “flat” pattern is characterized by an increased disjunction distance of the 90°–130° arch (7.5 ± 2.3 mm) and 140°–220° arch (7.8 ± 2.4 mm) with closer values of disjunction measured at the 230°–280° arch (7.0 ± 2.7 mm). The maximal disjunction is located at 140° on the ideal circumference of the valve, with a mean extension of 7.7 ± 2.3 mm. The Student’s *t*-test performed on this distribution was able to find significance only for the lower extent of disjunction in the anterior commissure when compared to P3 and P2 segment (*p* = 0.047). An overall difference in the extent and pattern of mitral lesions was identified in our retrospective analysis. The most frequent lesion identified in the commissural phenotype of disjunction was the chordal rupture (*n* = 12, 57%), while the presence of cleft-like indentation (*n* = 6, 29%) and bileaflet prolapse (*n* = 8, 38%) added additional complexity to the lesions’ set. Flat phenotype showed a marked tendency to bileaflet involvement (*n* = 9, 75%) with a reduced presence of both chordal rupture (*n* = 4, 33%) and cleft-like indentation (*n* = 2, 17%).

**TABLE 2:** Regional values of disjunction.

		Posterior mitral leaflet extension					
			
		Posterior commissure	P3	P2	P1	Anterior commissure	*P*-value
							
		60°–80°	90°–130°	140°–220°	230°–280°	290°–310°	
MAD (*n* = 33)	DD, mm	5.4 ± 2.2	7.3 ± 1.9	5.1 ± 3.0	5.7 ± 3.3	4.7 ± 2.9	< 0.05*
Commissural (*n* = 21)	DD, mm	5.4 ± 2.4	7.2 ± 1.8	3.6 ± 2.2	5.0 ± 3.4	4.1 ± 3.0	< 0.05**
Flat (*n* = 12)	DD, mm	5.5 ± 2.1	7.5 ± 2.3	7.8 ± 2.4	7.0 ± 2.7	5.8 ± 2.5	< 0.05^†^

Values are mean ± SD. *p=0,0005 for P3 versus posterior commissure; 0,001 versus P2; 0,02 verus P1 and 0,00007 versus anterior commissure. **p=0,007 for P3 versus posterior commissure; 0.0000001 versus P2; 0.01 versus P1 and 0,0002 versus anterior commissure. ^†^p=0.02 for P2 versus posterior commissure and p=0.04 for P3 versus posterior commissure. DD, Disjunction distance; MAD, Mitral annular disjunction.

### Quantitative analysis of annular structure and leaflets

Average measures of annular dimensions are shown in Table 3. The patients with a flat phenotype of disjunction showed an increased antero-posterior mitral diameter (43,3 ± 5.3 vs. 40,6 ± 4.3 mm), intercommissural width (54,4 ± 8.4 vs. 52,8 ± 5.0 mm), circumference (166 ± 26 vs. 159 ± 14 mm) and annular area (2,085 ± 616 vs. 1,862 ± 331 mm^2^), even if neither of this differences were deemed significative. Likewise, the surrogate equations which were used to convey the annular ellipticity and the non-planarian saddle-shape of the valve, failed to show a difference in this two phenotypes. All the morphological values were then indexed to body surface area and to end-diastolic left ventricular volume, when appropriate. Anyhow, in these two patterns of disjunction no significant difference was detected neither for the individual measurement nor for the indexed values.

**TABLE 3:** Annular and leaflets measurements.

			Distribution pattern		
				
		MAD (*n* = 33)	Commissural (*n* = 21)	Flat (*n* = 12)	*P*-value
**Annulus**					
Intercommissural width	mm	53.4 ± 6.2	52.8 ± 5.0	54.4 ± 8.4	0.17
Indexed intercommissural width	mm/m^2^	29.5 (26.2–30.5)	28.7 (25.9–30.7)	28.7 (26.7–30.7)	0.82
Anteroposterior diameter	mm	41.5 ± 4.7	40.6 ± 4.3	43.3 ± 5.3	0.17
Indexed anteroposterior diameter	mm/m^2^	22.7 ± 3.6	22.3 ± 3.4	23.5 ± 3.3	0.37
Circumference	mm	161 ± 19	159 ± 14	166 ± 26	0.34
Indexed circumference	mm/m^2^	88 (80–93)	86 (79–93)	88 (80–94)	0.67
Area	mm^2^	1,936 ± 447	1,862 ± 331	2,085 ± 616	0.23
Indexed area	mm^2^/m^2^	991 (878–1,207)	985 (876–1,097)	1,103 (893–1,223)	0.46
Height	mm	5.3 ± 1.5	5.1 ± 1.5	5.7 ± 1.4	0.35
Indexed height	mm/m^2^	2.9 ± 0.7	2.8 ± 0.7	3.1 ± 0.7	0.30
Annular height-to-intercommissural width ratio, %		9.9 ± 2.4	9.7 ± 2.7	10.4 ± 1.8	0.47
Intercommissural width-to-anteroposterior diameter ratio, %		129 ± 11	131 ± 12	126 ± 9	0.26
**Leaflet**					
Total leaflet area	mm^2^	2,534 ± 698	2,430 ± 584	2,740 ± 887	0.29
Indexed total leaflet area	mm^2^/m^2^	1,277 (1,088–1,563)	1,203 (1,083–1,517)	1,447 (1,131–1,637)	0.43
Leaflet area-to-annulus area ratio, %		130 ± 10	130 ± 11	131 ± 9	0.81
Prolapse volume	ml	6.2 (3.2–8.6)	4.6 (3.0–8.8)	6.8 (4.8–10.8)	0.29
Prolapse volume ratio		0.034 (0.027–0.072)	0.032 (0.024–0.058)	0.041 (0.031–0.075)	0.40

Values are mean ± SD or median (interquartile range) as appropriate; intercommissural width, anteroposterior diameter, circumference, annular area, height and total leaflet area are indexed (i) to BSA while prolapse volume is indexed to EDV. BSA, body surface area; EDV, end diastolic volume; MAD, mitral annular disjunction.

### Surgical outcomes

All patients successfully underwent surgical repair. In 10 patients, tricuspid valve repair was also performed, according to the current valvular indications (8). A minimally invasive thoracotomy was the preferred surgical approach in 27 out of 33 cases (81.8%). One patient required an early reoperation due to persistent bleeding, one patient required a second aortic cross-clamp to achieve a satisfying valvular repair. Mean cardiopulmonary bypass time was 126,9 ± 28.0 min, mean aortic cross-clamp time was 92.5 ± 23.6 min. All patients received prosthetic annuloplasty ring implantation; triangular leaflet resection was performed in 19 cases (58%), neochordae implantation was the preferred technique in 3 cases (9%), while a secondary order tendineuos chord transposition was performed in one patients. Regarding the 17 patients with a bileaflet extension of the prolapse, in 9 of them the pre-procedural evaluation showed a symmetric billowing of the leaflets without any local prolapsing prevalence, a marked disjunction with a vigorous thickening of the basal posterior myocardial wall along with the systolic attraction of both papillary muscles toward the atrium. No prevalence of a specific MAD phenotype was detected in this peculiar cluster of patients (commissural pattern, *n* = 5, 56% vs. flat pattern *n* = 4, 44%). No adjunctive technique, beside the implantation of a prosthetic ring, was performed in this subset of patients. A satisfying coaptation depth was achieved in all patients. No case of residual regurgitation more than trivial or MAD were detected at the latest follow-up. Quantitative valvular analyses and intraoperative data of the two different surgical populations are detailed in Tables 4, 5, respectively. The group of patients treated with isolated annuloplasty showed increased intercommissural width (57.1 ± 6.6 mm vs. 51.3 ± 5.9 mm), antero-posterior diameter (43.7 ± 5.4 mm vs. 40.7 ± 5.4 mm), circumference (172 ± 20 mm vs. 156 ± 18 mm), and annular area (2,184 ± 530 mm^2^ vs. 1,819 ± 410 mm^2^), while comparable values of annular height, annular height-to-intercommissural width ratio and intercommissural width-to-anteroposterior diameter ratio were measured and calculated. Among these annular measurements, a threshold for statistical significance was reached only by intercommissural width distance (*p* = 0.04) and circumference length (*p* = 0.04). Conversely, a comparison between leaflet deformity showed in the isolated annuloplasty group a significant increase in total leaflet area (3,044 ± 841 vs. 2,319 ± 514 mm^2^), prolapse volume [13.7 (6.4–15.7) vs. 4.4 (2.6–6.8) ml] and leaflet area-to-annulus area ratio (138 ± 10% vs. 128 ± 9%). Interestingly, the comparison of indexed values was able to find an additional statistical significance: besides the already detected parameters, the anteroposterior diameter was additionally found to be increased in the isolated annuloplasty group (*p* = 0.04) when compared to the patients who needed adjunctive repair techniques.

**TABLE 4:** Annular and leaflets measurements.

			Surgical technique		
				
		MAD (*n* = 33)	Complex (*n* = 24)	Isolated annuloplasty (*n* = 9)	*P*-value
**Annulus**					
Intercommissural width	mm	53.4 ± 6.2	51.3 ± 5.9	57.1 ± 6.6	0.03*
Indexed intercommissural width	mm/m^2^	29.5 (26.2–30.5)	27.5 (26.2–29.5)	31.3 (28.8–38.5)	0.01*
Anteroposterior diameter	mm	41.5 ± 4.7	40.7 ± 5.4	43.7 ± 5.4	0.19
Indexed anteroposterior diameter	mm/m^2^	22.7 ± 3.6	21.8 ± 2.6	24.7 ± 4.2	0.04*
Circumference	mm	161 ± 19	156 ± 18	172 ± 20	0.04*
Indexed circumference	mm/m^2^	88 (80–93)	84.0 (77.2–89.2)	94.5 (85.5–111.9)	0.02*
Area	mm^2^	1,936 ± 447	1,819 ± 410	2,184 ± 530	0.05
Indexed area	mm^2^/m^2^	991 (878–1,207)	964 (870–1,085)	1,170 (969–1,476)	0.06
Height	mm	5.3 ± 1.5	5.3 ± 1.3	5.6 ± 1.7	0.46
Indexed height	mm/m^2^	2.9 ± 0.7	2.7 ± 0.6	3.2 ± 0.9	0.16
Annular height-to-intercommissural width ratio, %		9.9 ± 2.4	10.0 ± 2.4	9.9 ± 2.8	0.94
Intercommissural width-to-anteroposterior diameter ratio, %		129 ± 11	128 ± 10	131 ± 13	0.49
**Leaflet**					
Total leaflet area	mm^2^	2,534 ± 698	2,324 ± 510	3,044 ± 841	0.01*
Indexed total leaflet area	mm^2^/m^2^	1,277 (1,088–1,563)	1,195 (1,066–1,465)	1,721 (1,213–2,136)	0.02*
Leaflet area-to-annulus area ratio,%		130 ± 10	128 ± 9	138 ± 10	0.003*
Prolapse volume	ml	6.2 (3.2–8.6)	4.5 (2.6–6.8)	13.7 (6.4–15.7)	0.002*
Prolapse volume ratio		0.034 (0.027–0.072)	0.031 (0.023–0.087)	0.087 (0.043–0.140)	0.003*

Values are mean ± SD or median (interquartile range) as appropriate; intercommissural width, anteroposterior diameter, circumference, annular area, height and total leaflet area are indexed (i) to BSA while prolapse volume is indexed to EDV. *p < 0.05 between the two surgical sets of patients. BSA, body surface area; EDV, end diastolic volume; MAD, mitral annular disjunction.

**TABLE 5:** Intraoperative data.

		Surgical technique		
			
	MAD (*n* = 33)	Complex (*n* = 24)	Isolated annuloplasty (*n* = 9)	*P*-value
Cardiopulmonary bypass time, min	127 ± 28	125 ± 30	131 ± 24	0.59
Aortic cross clamp time, min	92 ± 24	91 ± 23	95 ± 26	0.73
Prosthetic ring size, mm	36.0 (34.0–38.0)	36.0 (32.0–36.0)	38.0 (36.5–40.0)	0.04*

Values are mean ± SD or median (interquartile range) as appropriate. *p < 0.05 between the two surgical sets of patients.

## Discussion

In the landscape of DMVD, the present study is among the firsts (2, 5) to characterize the detailed circumferential distribution of MAD and its geometric and surgical implications. The coupled motion of MA and the left ventricular contraction is pivotal to achieve a proper coaptation and ensure valve continence, allowing a balanced distribution of stresses and tensions on the mitral complex. The first evidence of the absence of this relation in MAD was provided in the seminal work of Lee et al. (2). Using acquired data from real time 3-dimensional echocardiography, the authors unveiled the functional decoupling of the annular and ventricular motions, which leads to a paradoxical systolic annular dilatation and unsaddling of the annular shape. In their cohort of 42 patients with MAD, the analysis of the relation of this feature with valvular structure and function showed a more severe deformity of the leaflets and chordae tendineae, with the disjunction generally located adjacent to the prolapsed segments and with a maximal DD measured at P2 scallop in 47.6% of the subjects. While the findings in our cohort confirm the common prevalence (***n*** = 50, 58.8%) of MAD in the context of MMVD, in our analysis of the 3-dimensional annular anatomy we were able to identify two different patterns of disjunction: a commissural and a flat phenotype, with a maximal DD observed at the central region of the posterior annulus (P2 scallop) only in 7 cases. A crucial element of our study was the careful recognition of the so-called pseudo-MAD. In our cohort, it was possible to recognize the isolated presence of this entity in 24 patients (28%). Although the true-MAD prevalence is higher in our experience (58.8% of all the surgical patients with MMVD), the presence of the confounding pseudo-MAD was observed indeed even in the context of a truly disjuncted annulus. Actually, the two entities, already identified by Faletra et al. (6), coexisted in the same MA in one third of our analysis’ final cohort. Despite the increasing interest in the understanding of MAD, complex and comprehensive data of its distribution and implications are lacking. Our study provides rare insights into MAD phenotypes, allowing the identification of two major patterns with a higher prevalence of a commissural disjunction. Although MAD is constantly associated with profound annular alteration and marked leaflet redundancy, the feasibility of MVR is not impaired and only a small percentage of patients require a very complex repair technique. Our analysis identified a peculiar subgroup of patients in which the isolated annuloplasty may restore the physiological balance of mitral complex. These patients were those with the most increased annular dimensions and leaflet redundancy, as shown in Table 4. The disjointed annulus in these cases was the most remarkable of any subset analyzed, with the highest values of disjunction and leaflet deformity [peak disjuncted distance 10.6 ± 1.9 mm, prolapse volume 13,7 (6.4–15.7) ml and leaflet area 3,044 ± 841 mm^2^]. The disjunction in these cases was distributed in both commissural or flat patterns. This peculiar subgroup was rather identified because of their symmetric redundancy of both anterior and posterior leaflets and a peculiar dynamics. Doppler analysis showed a broad-based central regurgitant jet Supplementary Video 1 and surgical inspection confirmed in each case the diffuse myxomatous alteration of the leaflets. The marked muscular detachment from the normal annular anchoring resulted in a prominent, vigorous, basal bulging with an hyperkinetic aspect and significant thickening. This feature, recognized almost in every MAD patients, was interestly found to be reversible after surgical correction in the work of Essayagh et al. (9), with the evidence of restored diastolic wall thickness and systolic thickening. Another pivotal element of this mitral entity is the papillary muscles displacement toward the left atrium. Although an unequivocal and detailed measurement of the position of both papillary tips and an analysis of their contraction was not possible in the present study, all the patients chosen for an isolated annuloplasty repair showed this feature. In this group, surgical intervention involved an isolated non-restrictive annuloplasty (prosthetic ring mean size 37,5 ± 3,3 mm). Mean cardiopulmonary bypass time was 131 ± 24 min and mean aortic clamping time was 95 ± 26 min. When compared to the patients who underwent more complex valvular repair, this group showed an increased size of implanted prosthetic ring (37,5 vs. 35,1 mm), while no difference was observed in terms of surgical performance (cardiopulmonary bypass time 131 vs. 125 min, aortic clamping time 95 vs. 91 min).

## Clinical implication and future perspective

Our observations corroborate the early evidence (2, 5) of an uneven circumferential distribution of the disjuncted MA. Despite MAD is commonly diagnosed and measured in parasternal long axis view, our analysis showed that central regions of the posterior MA are less frequently the most disjuncted (22%), and therefore the long axis transthoracic evaluation of disjunction could be sub-optimal to estimate its prevalence, leading to a potential diagnostic bias. TEE, with its increased spatial resolution and definition, should be considered in our opinion a more appropriate exam to routinely and comprehensively assess the presence and extension of MAD, given its heterogeneous distribution. A growing body of evidence has suggested that disjunction prevalence in general population is not a rare finding, especially when imaging modalities with higher spatial resolution were used (5, 10). Although there has been an increased attention to MAD presence and extension, due to its association with MMVD and sudden cardiac death (4), a recent European survey on valvular heart disease clinical experience (11) reported that TTE is still the modality of choice to asses MAD, while cardiac magnetic resonance (CMR)–which is usually considered as gold standard among the different imaging techniques (12–14)–is only adopted by 29% of the respondents. On the other hand, due to its intrinsic bi-dimensional nature, dedicated and standardized protocols should be validated and implemented in order to replicate a careful analysis of MAD extension along every mitral segment. In the spectrum of MMVD, a complex plethora of lesions and leaflet’s abnormalities are known to reduce the feasibility of a durable repair leading to challenging surgical scenarios. Nevertheless, even in the context of advanced BD, the main annular feature reported is the mere dilatation of its diameters, along with the loss of its typical saddle-shape. With its high prevalence and heterogeneity, MAD is usually underestimated and rarely comprehensively evaluated in pre-operative work-ups. Very few attempts were made in order to surgically address this feature (15) which is often neglected or forgotten. In the landscape of complex MVR, the symmetric bileaflet prolapse represents a highly selected cohort of patients in which MAD could be identified as an active pathogenetic mechanism (16). The surgical correction of this annular abnormality with an isolated annuloplasty is a high-feasible and low-risk procedure (17–19). Moreover, in similar cohort of patients with MAD, MVR showed to be effective not only to provide the restoration of a proper coaptation, but also to restore a normal systolic thickness and pattern of myocardial wall contraction (9). This positive remodeling, along with further analysis of the prolapsing volume’s impact on mitral regurgitation pathophysiology (20, 21) and the potential surgical benefit exerted on the arrhythmic profile (22), should be pivotal elements to take into account for the lifetime management of this peculiar valvular disease.

## Study limitation

In order to increase the understanding of MAD 3-dimensional anatomy, we developed and enhanced in our study a complex approach, which integrates the 3-dimensional echocardiographic analysis of the disjunction and the quantitative modeling of the mitral valve. Further studies are needed in order to validate the proposed workflow, which is still remarkably time-consuming, and to find an agreement about validated measuring methods of MAD extent. The retrospective nature of the study, the small sample size and the short term results of our repaired mitral valves are major limitations we were not able to avoid in the present work. A prospective, multicentric design, results’ reproducibility in larger cohorts of patients and an appropriate follow-up are also mandatory elements to look for in the following years in order to deepen the knowledge about MAD.

## Conclusion

Mitral annular disjunction should be considered an intrinsic component of the myxomatous disease spectrum, with its complex and heterogeneous morphology. TEE should be considered a mandatory exam to routinely assess the presence and extension of MAD, along with its prevalent phenotypes which can be easily addressed without an increased surgical challenge.

## Data availability statement

The original contributions presented in the study are included in the article/Supplementary material, further inquiries can be directed to RB, raoul.biondi@gmail.com.

## Ethics statement

The studies involving human participants were reviewed and approved by the Institut Mutualiste Montsouris. Written informed consent for participation was not required for this study in accordance with the national legislation and the institutional requirements.

## Author contributions

RB and KZ: conception and design. RB, CD, SR, and AB: analysis and interpretation of clinical images and data. RB, KZ, NA, GR, NK, and SR: drafting of the manuscript or revising it critically for important intellectual content. All authors contributed to the article and approved the submitted version.
